# Label-Free Imaging of Melanoma with Confocal Photothermal Microscopy: Differentiation between Malignant and Benign Tissue

**DOI:** 10.3390/bioengineering5030067

**Published:** 2018-08-15

**Authors:** Takayoshi Kobayashi, Kazuaki Nakata, Ichiro Yajima, Masashi Kato, Hiromichi Tsurui

**Affiliations:** 1 Advanced Ultrafast Laser Research Center and Brain Science Inspired Life Support Research Center, University of Electro-Communications, 1-5-1 Chofugaoka, Chofu, Tokyo 182-8585, Japan; washinkt@gmail.com; 2 JST, CREST, 5 Sanbancho, Chiyoda-ku, Tokyo 102-0075, Japan; 3 Department of Physics, Tokyo University of Science, Kagurazaka 1-3, Shinjuku-ku, Tokyo 162-8601, Japan; 4 Department of Electrophysics, National Chiao-Tung University, 1001 Ta Hsinchu Rd., Hsinchu 300, Taiwan; 5 Research Center for Water Frontier Science and Technology, Tokyo University of Science, Kagurazaka 1-3, Shinjuku-ku, Tokyo 162-8601, Japan; 6 Department of Occupational and Environmental Health, Graduate School of Medicine, Nagoya University, 65 Tsurumai-cho Showa-ku, Nagoya, Aichi 466-8550, Japan; yajimai@med.nagoya-u.ac.jp (I.Y.); katomasa@med.nagoya-u.ac.jp (M.K.); 7 Department of Pathology, Juntendo University School of Medicine, 2-1-1 Hongo, Bunkyo-ku, Tokyo 113-8421, Japan; tsurui@juntendo.ac.jp

**Keywords:** photothermal microscopy, label-free imaging, melanoma, texture analysis

## Abstract

Label-free confocal photothermal (CPT) microscopy was utilized for the first time to investigate malignancy in mouse skin cells. Laser diodes (LDs) with 405 nm or 488 nm wavelengths were used as pumps, and a 638 nm LD was used as a probe for the CPT microscope. A Grey Level Cooccurrence Matrix (GLCM) for texture analysis was applied to the CPT images. Nine GLCM parameters were calculated with definite definitions for the intracellular super-resolved CPT images, and the parameters Entropy, Contrast, and Variance were found to be most suited among the nine parameters to discriminate clearly between healthy cells and malignant cells when a 405 nm pump was used. Prominence, Variance, and Shade were most suited when a pump wavelength of 488 nm was used.

## 1. Introduction

Malignant melanoma (MM) is one of the most common cancers worldwide. It has a favorable prognosis only if the affected area is removed at an early stage. MM reportedly causes the large majority of skin cancer deaths despite the fact that it accounts for <2% of skin cancer cases [1]. The incidence of MM has been increasing for >30 years [2] and one of its most ominous characteristics is its high propensity to produce distant metastases, because it can get disseminated throughout the body through lymphatic and hematogenous spread. For this reason, early detection and treatment of MM are crucial life-saving measures [3]. Although dermoscopy is a powerful diagnostic technique [4] and the ABCDE (abbreviation for asymmetrical shape, border, color, diameter, and evolution) rule provides a guide to the identification of involved areas [5], pathological examination is even now the gold standard for MM diagnosis. However, diagnosis remains subjective and highly reliant on the skill level of the pathologist. Interobserver reproducibility of MM diagnosis varies even among experts.

Fractal analysis has been developed as a strategy to improve diagnostic reliability. This method is based on the calculation of the fractal dimension (FD) of the structure of MM cells and their distribution [6,7,8,9,10,11]. Although this is a very attractive idea, the method has three drawbacks. First, the technique is based on self-similarity, namely, having its reduced image in itself recursively; therefore, if no self-similarity is present in the structure, the arbitrarily defined FD is difficult to connect to the structural features being discussed in the paper. Second, the FD is based on the calculation of the line length or area of the covered region, and changes in its value are then visualized by changing the analyzed domain. This procedure limits the ratio between the minimal size of evaluation and the total size, resulting in limited spatial resolution and spatially dependent information. Finally, marker-free phenotyping of tumor cells by fractal analysis of reflection interference contrast microscopy (RICM) images results in a very small difference in FD values between the different types of MM cells [12]. For example, two types of MM cells in one study had FD values of 1.353 ± 0.004 and 1.312 ± 0.005, which corresponds to only a 3.08% difference in the average value of the two groups [12]. Even though these authors claimed that the standard deviations (SDs) were as small as 0.005 and 0.004, the FDs in Figure 5 of their paper showed nearly 80% overlap, while their RICM images showed very clear visual differences in the apparent structural features between the two types (Figure 5A of the paper). This means that, even when the image patterns are quite different between two images, similar FDs can be obtained, indicating that the method is difficult to apply to clinical diagnosis. This disadvantage forms the basis of our motivation to find a more reliable and useful diagnostic application of textural structure to skin cancer cells in the present paper.

Melanin carries information about the metabolism and location of melanocytes and melanogenesis; therefore, the melanin distribution could act as a marker for MM [13,14]. The two dominant types of melanin (eumelanin and pheomelanin) absorb a large cross section of visible light without substantial fluorescence emission [15], resulting in the difficulty in the imaging of MM distribution. However, the non-fluorescent property of melanin enables even more sensitive imaging of MM by employing photothermal (PT) microscopy (PTM), which is the main subject of the present paper.

PTM, which is based on the detection of changes to probe light intensity by thermal lensing due to local heating of the sample by absorption of the laser light, has demonstrated potential for biological imaging and clinical application. The key advantages of PTM are its high sensitivity and lack of a requirement for staining [16,17,18,19,20,21]. It facilitates the real-time, high-resolution imaging of nanometer-sized absorbers buried among light scatterers with a high signal-to-noise ratio [19,22,23]. However, the PT signal intensity in normal PTM has two extremes in the axial direction [24], which introduces distortions resulting in limited axial resolution of three-dimensional PT images. Confocal PTM (CPTM), which has a detection scheme similar to that of confocal microscopy, can help to ameliorate this drawback and improve the axial resolution [24]. Our group has used CPTM to study super-resolution microscopic images of neurons in mouse brains [25,26] and mouse skin MM [27].

In our previous paper [28], we developed a CPTM system and applied it to examine the features of melanin aggregates of both nevus and MM cells in thick (10–15 μm) specimens of a melanoma model mouse. The *ret* gene is a receptor tyrosine kinase type oncogene, and *rfp* is a fusion of 5′-half of ret gene and a finger structure probably capable to bind DNAs. RET-transgenic mice of the 304/B6 (RET-Tg) line develop benign melanocytic tumors and MM in a stepwise manner, and they are widely used for the study of melanoma genesis [29,30,31]. Obtained 3D images were analyzed to characterize the features of the melanin aggregates such as the density, size, and the surface FD. Even though the differences of those features between the two cell types were clearly demonstrated, we were not able to evaluate very accurately its diagnostic capability because of the limited image quality due to slow stage with which photodegradation during one frame imaging. The photo-damage was inevitable to get images with intensity enough to high S/N but it induced signal intensity reduction in the later stage of the one-frame imaging process.

In this study, we made an improvement upon the CPTM technique and applied it for noninvasive label-free imaging of MM in the mouse model previously used for the purpose of future application to humans. The scanning method was changed from piezo-driven stage to galvanometer, hence the wider swept area from 20 × 20 μm^2^ to 72 × 72 μm^2^ and the shorter scanning period from 3 ms to 20 μs. This enabled to solve the problem in the previous study which hampered us to get enough quality images. The performance of the analytical system was tested with a sample of 20 nm gold nanoparticles. Using the PT imaging data, we then analyzed the structural properties of MM and nevus cells to be compared using the GLCM method [32,33,34,35,36,37,38]. We calculated nine different parameters: angular second moment (ASM), Contrast, Correlation, Entropy, inverse difference moment (IDM), Homogeneity, Prominence, Shade, and Variance. These textural parameters were determined by analyzing relevant regions of interest on 12 two-dimensional PT images obtained at two different positions in three tissue sections containing nevus and MM cells. The details of this analysis are described in the following experimental section. Our method provides an objective evaluation independent of the experience, skill, and knowledge of individual medical doctors, and prognostication at each occasion of the pathological diagnosis. Thus, GLCM calculation provides a quantitative indicator that may become a “standard” in the future by the accumulation of cases in real clinical settings from various individuals with a variety of experiences. We first applied difference criterion (DIF) analysis and receiver operating characteristic (ROC) curve analysis. The analyses provided the results that Entropy, Contrast, and Variance were most suited for the discrimination between MM and nevus when 405 nm excitation was used, and Prominence, Variance, and Shade were most at 488 nm excitation. Second, we defined a new index, a “clearness discrimination parameter” (DISC), for the discrimination between nevus and MM cells. This index suggested that Entropy and Homogeneity in the case of the 405 nm pump, and Entropy and Prominence in the case of the 488 nm pump were the two most suited among the nine parameters for discrimination between nevus and MM cells in a mouse. This research received no external funding.

## 2. Materials and Methods

### 2.1. Mice

Nevus and MM samples, which were diagnosed in advance by the specially trained pathologist, in transgenic mice (RET-Tg) carrying constitutively activated RFP/RET, a hybrid oncogene between RFP and c-RET [31], were prepared and used for analysis. The Animal Care and Use Committee (approval no. 270108 in Juntendo University) and the Recombination DNA Advisory Committee (approval no. 13-7626-55 in Juntendo University) approved this study.

### 2.2. Experimental Setup and Image-Taking Procedure

**Figure 1:**
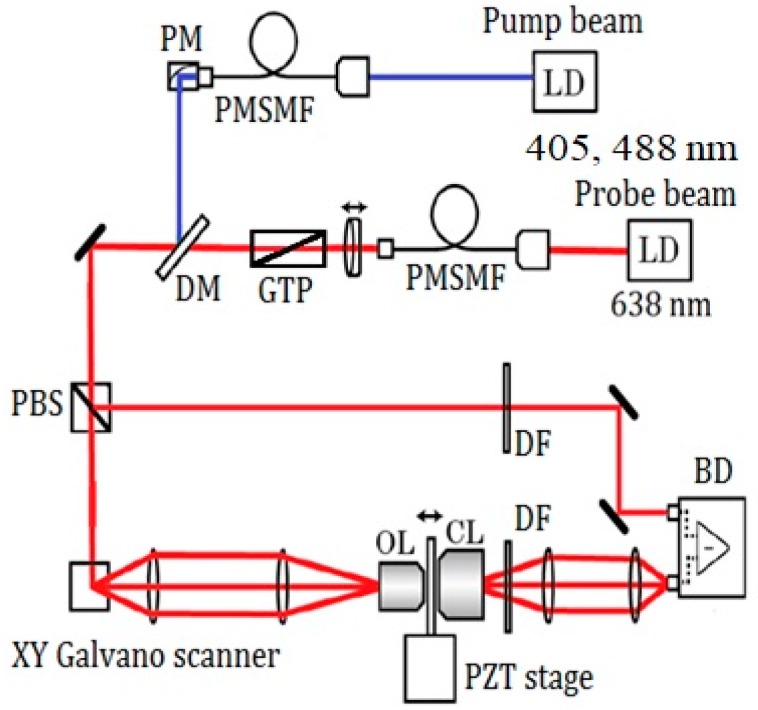
Scheme of the experimental setup. LD: laser diode, PMSMF: polarization maintaining, single-mode fiber, PM: parabolic mirror, GTP: Glan-Thompson polarizer, DM: dichroic mirror, PBS: polarizing beam splitter, OL: objective lens, CL: condenser lens, DF: dielectric multi-layer filter, BD: balanced detector, PZT stage: piezo driven stage.

Figure 1 shows the scheme of the experimental setup. Two sets of pump wavelengths are used in the experiment. A 405 nm laser diode (LD) (NDV4316; Nichia, Tokushima, Japan) and a 488 nm LD (L488P60; Thorlabs, Newton, NJ, USA) were used for the pump laser. A 638 nm LD (ML520G55; Mitsubishi, Tokyo, Japan) was used for the probe. The pump laser was modulated at 100 kHz using signal generators, and one of the two LDs was introduced to a beam collimator. After combination of the pump and probe beams using a dichroic mirror, the probe was split by a polarized beam splitter for balance detection and then directed to a galvano mirror (VM500PLUS; GSI Group, Bedford, MA, USA). A 4F optical system was located near the sample and objective lens (MPLFNF 40× with numerical aperture of 0.75; Olympus, Tokyo, Japan). Two-dimensional images of the samples were obtained by scanning laser beams with the galvano mirror in the X–Y plane. The irradiation powers of the pump and probe lasers were 300 μW (405 nm), 1.5 mW (488 nm), and 3 mW, respectively. One of the two pump lasers was used at one time. An auto-balanced detector (New Focus Nirvana; Newport Inc., Irvine, CA, USA) composed of two sets of a photodiode and a lock-in amplifier (7270 Signal Recovery; Ametek, Berwyn, PA, USA) was used to remove the noise from the probe. The frame size of the image was 600 × 600 pixels, which corresponds to a 72 × 72 μm^2^ area. The lock-in amplifier sensitivity was 1 mV, and the time constant was 20 μs per point. For the GLCM analysis, a four-section tiled area (18 × 18 μm^2^ out of 36 × 36 μm^2^) in the X–Y plane was imaged for each 72 × 72 μm^2^ image. We have analyzed 48 images of 18 × 18 μm^2^ area in total for both nevus and MM samples.

At the image obtaining process, we selected the areas to be investigated based on the following strategic criteria. Because the intensity of the PT signal varies with different areas, we selected those in which the obtained PT signals were strong enough to provide a high signal-to-noise ratio. In practice, we selected areas in which strong pigmentation was recognized.

The aim of the present study is to provide a non-specialist of the pathology of melanoma with a tool to distinguish between MM and nevus. The agreement of the classification of them in the present research was consistent with the classification performed by another specialist.

## 3. Results

**Figure 2:**
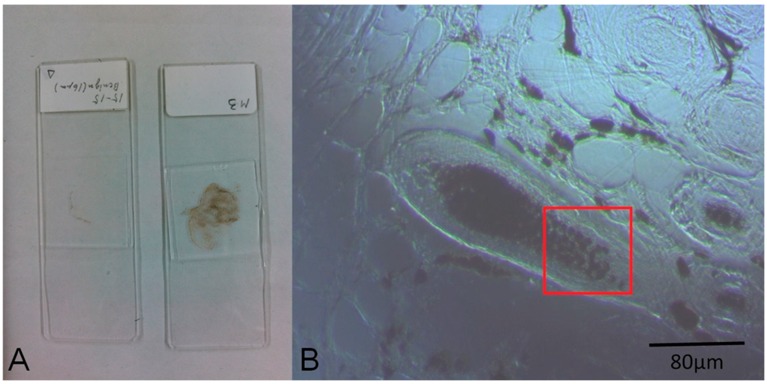
(**A**) Photo of the prepared specimens; (**B**) Charge coupled device (CCD) image of a nevus (10× objective lens). The red square area corresponds to the range of the photothermal (PT) image of Figure 3B.

**Figure 3:**
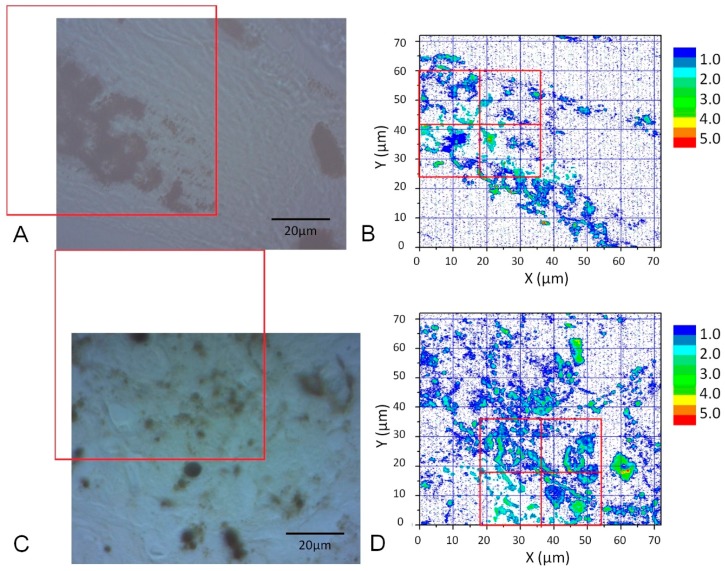
CCD images of a nevus (**A**) and malignant melanoma (MM) (**C**) (40× objective lens) and corresponding PT images (**B**,**D**) from the red squares obtained with a 405 nm pump. Four equal size (18 × 18 μm^2^) areas (red squares in (**B**,**D**)) were used for Grey Level Cooccurrence Matrix (GLCM) analysis.

**Figure 4:**
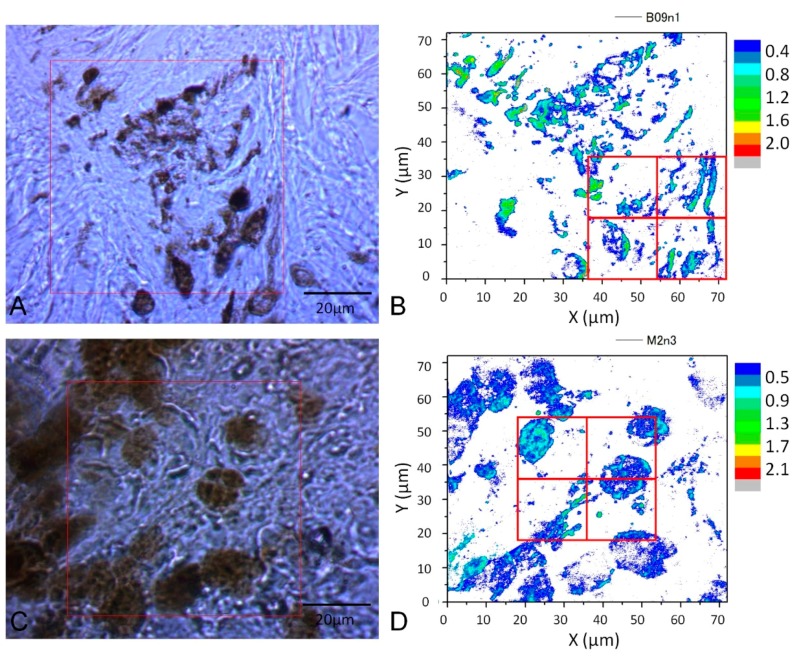
(**A**): CCD image and PT image of a nevus sample (40× objective lens)); (**C**): MM (40× objective lens) with 488 nm pump. (**B**,**D**): four equal size (18 × 18 μm^2^) areas segmented from the four red square areas in the PT images are used for GLCM analysis.

The images of cell samples of about 1 µm on 1 mm thick microscope slides are shown in Figure 2, Figure 3 and Figure 4. Figure 2 shows a photograph of cutaneous tissues taken from mice with MM. Microscopic regions of 72 × 72 µm^2^ in the PT images were selected from the images for analysis as shown in Figure 3 and Figure 4. The two bright field images in Figure 3 are obtained with charge coupled device (CCD) (DCC1645C, Thorlabs, NJ, USA) for the nevus sample (top left) and the MM (bottom left) with a 40× objective lens. The wavelength and power of excitation of the LD was 405 nm and 0.3 mW, respectively. Top and bottom right are the PT images using a 405 nm pump (600 × 600 pixels, 120 nm/pixel) of nevus and MM cells, respectively. Red squares in the bright field images show the 72 × 72 μm^2^ areas in the PT images. Four equal size (18 × 18 μm^2^) areas segmented from the red square (36 × 36 μm^2^) portion in the PT images were used for GLCM analysis. In Figure 4, top left and top right are the bright field CCD images of nevus samples and MM samples, respectively, obtained with a 40× objective lens and with 488 nm excitation under the same condition as the ones in Figure 3. The top and bottom right images are the corresponding PT images (600 × 600 pixels, 120 nm/pixel) of nevus and MM samples, respectively. Red squares in the bright field images show the 72 × 72 μm^2^ areas of the PT images. Four equal size (18 × 18 μm^2^) areas were segmented out of the four red square areas in the PT images and used for GLCM analysis. Twelve areas from the PT images were evaluated and 48 calculation regions were selected for both 405 nm and 488 nm excitation.

**Figure 5:**
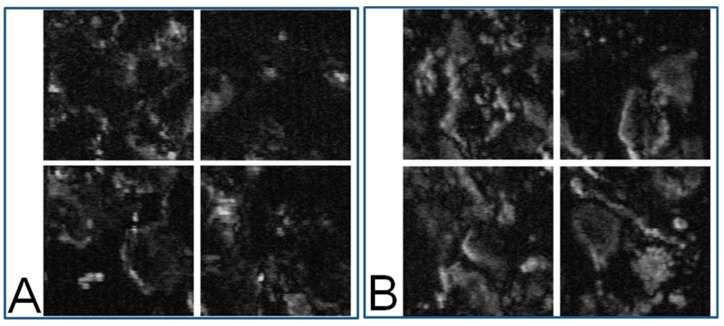
(**A**): Eight-bit gray-level PT image of a nevus sample (40× objective). The four images correspond to the four segmented areas represented by the four red squares in Figure 3B; (**B**): MM sample (40× objective). The four images correspond to segmented areas separated by the four red squares in Figure 3D.

**Figure 6:**
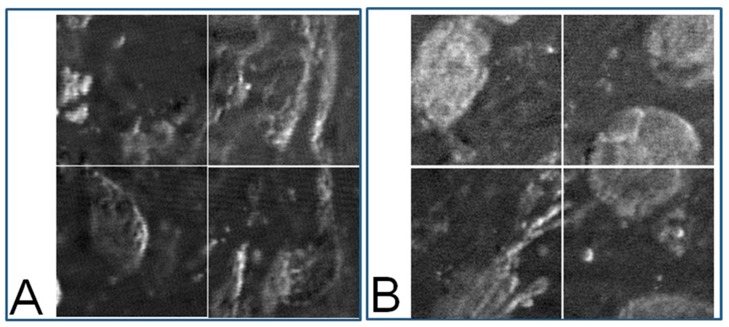
(**A**): Eight-bit gray level PT image of a nevus sample (40× objective). The four images correspond to the four segmented areas represented by the four red squares in Figure 4B; (**B**): MM sample (40× objective). The four images correspond to the four segmented areas represented by the four red squares in Figure 4D.

The textural structure of the images of the mouse skin samples containing both nevus and MM cells taken with the PT imaging method were analyzed by GLCM. The nine parameters were calculated as shown below. The areas of imaging data within the red lines in Figure 3 at 405 nm excitation were analyzed by the GLCM method, and the 8-bit level gray level intensity distribution of the PT signal is shown in Figure 5. Twelve images of 72 × 72 μm^2^ to multiple samples of excitation at 488 nm for both nevus and MM samples were also obtained. The areas of imaging data within the red lines in Figure 4 (left) at 488 nm excitation were analyzed by this method, and the 8-bit level gray level intensity distribution of the PT signal is shown in Figure 6. Four sets of 18 × 18 μm^2^ areas (shown in Figure 3 and Figure 4) with higher intensity out of the 12 images were chosen and analyzed using the GLCM analysis. There were total of 48 images with an area of 18 × 18 μm^2^ from both nevus and MM. This provided sufficient data at two sets of pump wavelengths to ensure statistical reliability. As shown in the bright field images, the sample areas are selected out of various parts of skin. For this technique to become a standard method, many more samples from a larger number of patients are required. However, as discussed below, we discovered that a few of the nine GLCM parameters clearly showed the ability to discriminate between nevus and MM, and can hopefully be used as criteria for pathological diagnosis.

Hereafter, the formalism of GLCM is described [39,40] by showing nine parameters out of the most frequently used indexes.

In all of the following formulas, *P* (*i*, *j*) stands for the (*i*, *j*)th entry or value in a normalized GLCM.

(1) $$ASM=\sum_{i=0}^{G-1}\sum_{j=0}^{G-1}{\left\{P(i,j)\right\}}^{2}$$

(2) $$Contrast=\sum_{|i-j|=0}^{G-1}|i-j{|}^{2}\{\sum_{i=1}^{G}\sum_{j=1}^{G}P(i,j)\}$$

(3) $$Correlation=\sum_{i=0}^{G-1}\sum_{j=0}^{G-1}\left[\frac{\left\{i\times j\}\times P(i,j)-\{\mu_{x}\times \mu_{y}\right\}}{\sigma_{x}\times \sigma_{y}}\right]$$

(4) $$Entropy=-\sum_{i=0}^{G-1}\sum_{j=0}^{G-1}P(i,j)\times \log(P(i,j))$$

(5) $$Homogeneity=\sum_{i=0}^{G-1}\sum_{j=0}^{G-1}\frac{1}{1+|i-j|}P(i,j)\;$$

(6) $$IDM=\sum_{i=0}^{G-1}\sum_{j=0}^{G-1}\frac{1}{1+{\left(i-j\right)}^{2}}P(i,j)$$

(7) $$Prominence=\sum_{i=0}^{G-1}\sum_{j=0}^{G-1}{\left(i+j-\mu_{x}-\mu_{y}\right)}^{4}P(i,j)$$

(8) $$Cluster\;Shade=\sum_{i=0}^{G-1}\sum_{j=0}^{G-1}{\left(i+j-\mu_{x}-\mu_{y}\right)}^{3}P(i,j)$$

(9) $$Variance=\sum_{i=0}^{G-1}\sum_{j=0}^{G-1}{\left(i-\mu \right)}^{2}P(i,j)$$

(10) $$\mu_{x}=\sum_{i=0}^{G-1}i\sum_{j=0}^{G-1}P(i,j)$$

(11) $$\mu_{y}=\sum_{j=0}^{G-1}j\sum_{i=0}^{G-1}P(i,j)$$

where $\mu_{x}=\mu_{y}=\mu$ for a symmetric matrix.

**Figure 7:**
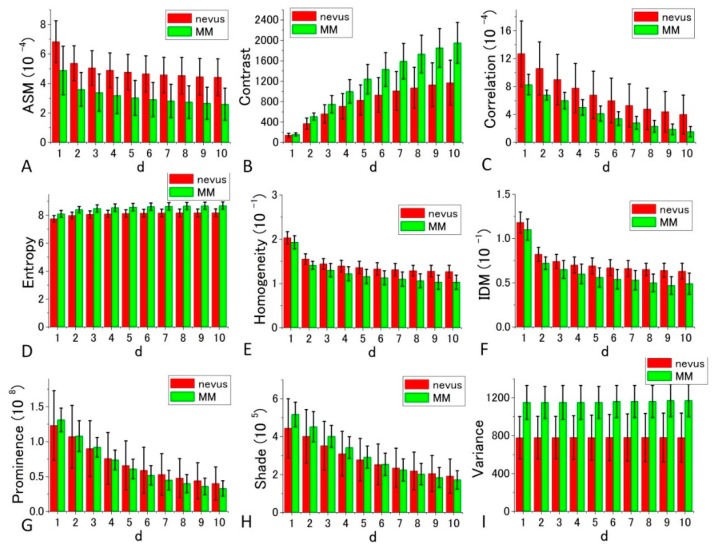
Histograms of nine calculated GLCM parameters (angular second moment (ASM), Contrast, Correlation, Entropy, Homogeneity, inverse difference moment (IDM), Prominence, Shade, Variance) for nevus (red bar), and MM samples (green bar) in 405 nm excitation. *p*-values of the *t*-test for all the parameters at *d* = 10 are, 6.72 × 10^−11^, 1.16 × 10^−10^, 2.71 × 10^−6^, 2.93 × 10^−27^, 2.36 × 10^−14^, 3.69 × 10^−13^, 0.374616, 0.071748, and 2.86 × 10^−7^ in the order of the panels, respectively.

**Figure 8:**
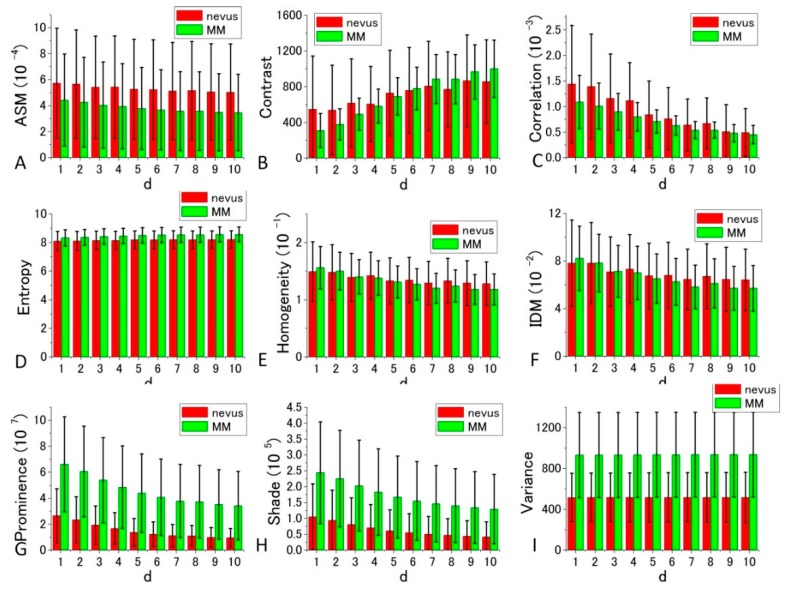
Histograms of nine calculated GLCM parameters (ASM, Contrast, Correlation, Entropy, Homogeneity, IDM, Prominence, Shade, Variance) for nevus (red bar), and MM samples (green bar) in 488 nm excitation. *p*-values of *t*-test for all the parameters at *d* = 10 are, 0.007765, 0.040389, 0.308247, 0.001409, 0.067924, 0.069963, 3.37 × 10^−8^, 1.65 × 10^−6^, and 2.61 × 10^−8^ in the order of the panels, respectively.

Figure 7 and Figure 8 depict bar charts of the nine averaged calculated distance parameters *d* = 1–10 (corresponding to the shift distance 120–1200 nm of the image in GLCM calculations) [39,40] and their standard deviations (SDs). In the graph, the bar is the SD of corresponding parameters calculated for the four sets of the image data shown in Figure 5 and Figure 6.

The features shown in Figure 7 and Figure 8 are summarized as follows.

The GLCM analysis of the PT images obtained at 405 nm excitation showed that the extent of the difference of nevus–MM distribution depends on the value *d*, and as it increases from 1 to 10, some parameters (namely Contrast, Entropy, Homogeneity and IDM) are considerably increased, whereas others (Correlation and Variance) showed very little change. As a whole, *d* = 10 provided the best separation, and among the nine parameters, Contrast, Entropy and Variance showed large differences between the means of nevus and MM cells, and they were almost equal to the sum of their SD values (ratios are between 0.9 and 0.95).

GLCM analysis of the PT images obtained by 488 nm excitation was also performed. Among the nine parameters, Prominence, Shade, and Variance were well separated between the nevus and MM cells compared with the other parameters. In these cases, the ratio of the difference between the two cell types to the sum of the values of SDs at *d* = 10 were 0.737, 0.635, and 0.557, respectively. The ratios in the other parameters were smaller than 0.31. To obtain suitable parameters for the identification of MM, we analyzed the data with a commonly used diagnostic method, including the parameters sensitivity, specificity, positive predictive value, and negative predictive value.

**Figure 9:**
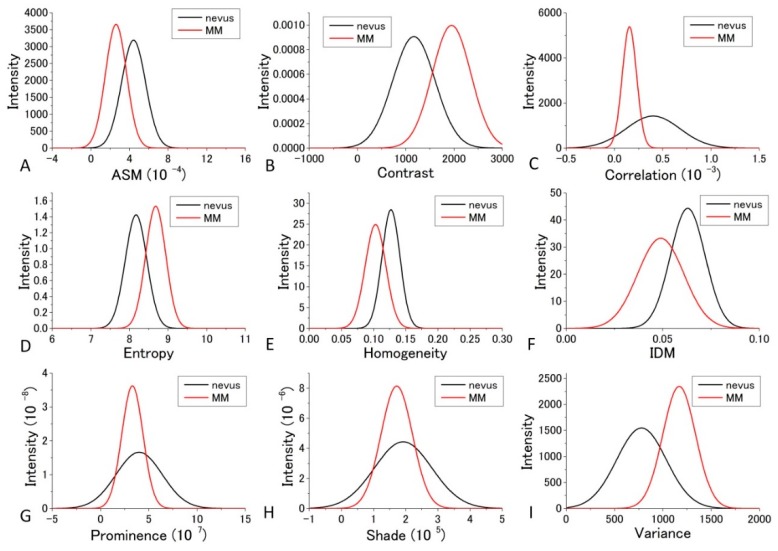
Gaussian distribution of the parameters fitted to the observed values of the parameters at 405 nm excitation.

**Figure 10:**
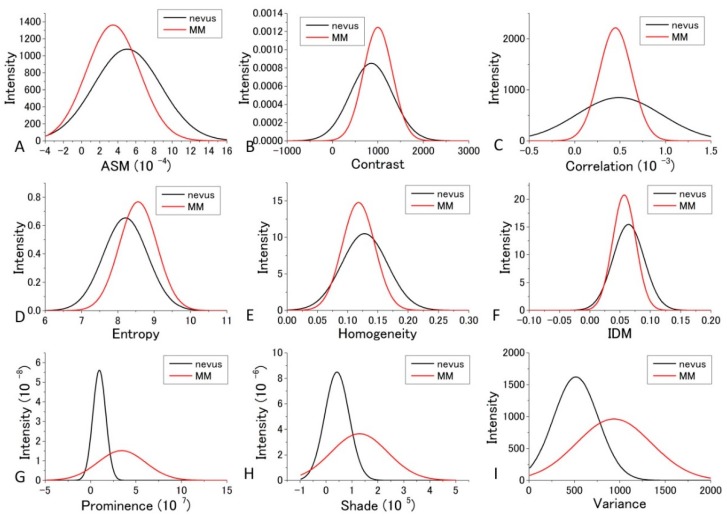
Gaussian distribution of the parameters fitted to the observed values of the parameters at 488 nm excitation.

For the diagnosis, we used Figure 7 and Figure 8 for each GLCM parameter for *d* = 10. We obtained the Gaussian curves fit to the data with the peaks and the widths being the mean and the SD values respectively in the histograms for *d* = 10 in Figure 7 and Figure 8 for each GLCM parameter; these data are shown in Figure 9 and Figure 10.

The differences in the parameters between malignant and benign can be discriminated even though it may not provide good enough discrimination for some parameters. We call this diagnosis the GLCM-DIAG method. The results of the discrimination parameters are listed for sensitivity (Sn), specificity (Sp), accuracy (AC), positive likelihood ratio (LR+), negative likelihood ratio (LR−), positive predictive value (PPV) and negative predictive value (NPV) in Supplementary Tables S1 and S2 using the data shown in Figure 9 and Figure 10 in the cases of 405 nm and 488 nm excitation, respectively. These figures show that the relative positions and widths of the two excitation wavelengths are quite different from each other. This is discussed later.

As shown in Supplementary Table S1, Variance, Entropy, and Contrast had large AC and LR+ values and a small LR− value, indicating that these three GLCM parameters are more reliable than the other GLCM parameters at 405 nm excitation. In the case of 488 nm excitation, as tabulated in Supplementary Table S2, Prominence, Shade, and Variance had large AC and LR+ values and a small LR− value, indicating that these three GLCM parameters are more reliable than the other GLCM parameters.

The distribution profiles calculated for the nine GLCM parameters at both 405 nm and 488 nm excitation are shown in Figure 9 and Figure 10.

To obtain suitable parameters for the identification of MM, we defined the difference criterion (DIF) as follows:

(12) DIF=|(PM−PN)|/[(DM+DN)/2]

where PM and PN are the parameter values of the MM and nevus cells, respectively, and DM and DB are the SDs of PM and PN for the four sets of imaging data. The rank orders of the magnitude and absolute values of DIF for the nine parameters are shown in Supplementary Tables S3–S6, respectively. Entropy, Variance, and Contrast were still ranked within the top three positions when *d* is near 10 in the 10 different *d*-value sets. Therefore, these three parameters (Entropy, Variance, and Contrast) are likely to be suited for the discrimination between nevus and MM cells.

From the above orders of the DIF values in Supplementary Tables S3 and S5, it can be concluded that Entropy, Contrast, and Variance are most suited for the discrimination between MM and nevus cells in 405 nm excitation. For Entropy and Contrast, the distance parameter *d* = 10 gives the highest DIF of 1.879 and 1.847, respectively, and for Variance, *d* = 2 produced the largest value, although it was not substantially larger than those of other values of *d*. The probabilities of correct identification of nevus cells were 39.49%, 39.21%, and 39.49% for Entropy (*d* = 10), Contrast (*d* = 10), and Variance (*d* = 10), respectively, while the probabilities of correct identification of MM cells were 39.49%, 39.21%, and 39.49% for Entropy (*d* = 10), Contrast (*d* = 10), and Variance (*d* = 10), respectively. These values are much more accurate than those obtained by fractal analysis [6,7,8,9,10,11].

From the orders of DIF values in Supplementary Tables S4 and S6, it can be concluded that Prominence, Variance, and Shade are most suited for the discrimination between MM and nevus cells in 488 nm excitation. For Shade and Variance, the distance parameter *d* = 10 gave the highest DIF values of 1.121 and 1.273, respectively, while for Prominence, *d* = 9 produced the highest value, although the difference between DIF at other values of *d* was very small. The probabilities of correct identification of nevus cells are 38.9%, 33.3%, and 33.6% for Prominence (*d* = 10), Shade (*d* = 10), and Variance (*d* = 10), respectively. The probabilities of correct identification of MM cells are 38.9%, 33.3%, and 33.6% for Prominence (*d* = 10), Shade (*d* = 10), and Variance (*d* = 10), respectively. These values are much better than those obtained by fractal analysis [6,7,8,9,10,11].

These findings indicate that the GLCM parameter method, especially GLCM-DIF analysis, is a simple and useful method for the identification of suitable parameters for differentiation between different stages of cancers and detection of various types of disease that alter cell structure.

**Figure 11:**
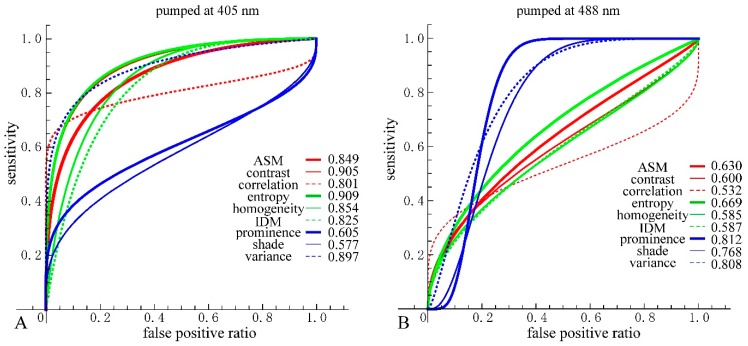
(**A**) Receiver operating characteristic (ROC) curves and area under the curve (AUC) for the nine parameters at 405 nm excitation. For consistency, directions of threshold-parameters scanning were set from nevus to MM. Nevus cells were considered as positive and MM cells as negative; (**B**) ROC curves and AUC for the nine parameters at 488 nm excitation.

We then performed receiver operating characteristic (ROC) curve analysis based on those Gaussian curves. A ROC curve is commonly used to evaluate the diagnostic ability of a test. When a threshold parameter used in the system classifying examinees into two groups, positive and negative for some features, this curve is plotted as the sensitivity against the false positive ratio. As shown in Figure 7 and Figure 8, *d* = 10 provides the best performance for all nine GLCM parameters in both cases pumped at 405 nm and pumped at 488 nm. We plotted ROC curves for the nine parameters (Figure 11A,B) based on the Gaussian curves (Figure 9 and Figure 10). The area under the curve (AUC) is an indicator of the diagnostic ability; >0.9, 0.7~0.9, and <0.7 correspond to high accuracy, moderate accuracy and poor accuracy, respectively. As shown in Figure 11A, Entropy, Contrast and Variance show high AUCs, namely 0.909, 0.905 and 0.897, respectively. These values indicate that those parameters provide highly accurate methods to distinguish nevus and MM cells. In the case of 488 nm excitation, the AUCs of Prominence, Variance and Shade are 0.812, 0.808 and 0.768, respectively, indicating worse performance than 405 nm excitation. Those results, both at 405 nm and 488 nm excitation, agreed with those of the DIF analysis.

## 4. Discussion

**Figure 12:**
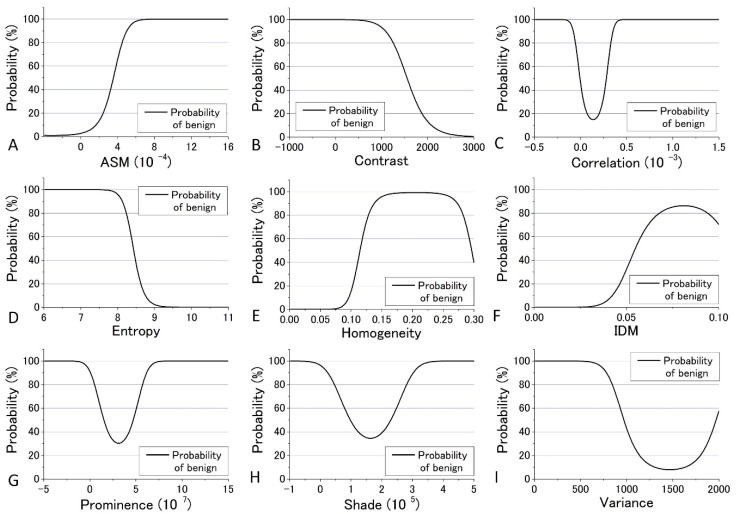
Probability of benign plotted against the parameter values for the distance *d* = 10 at 405 nm excitation.

**Figure 13:**
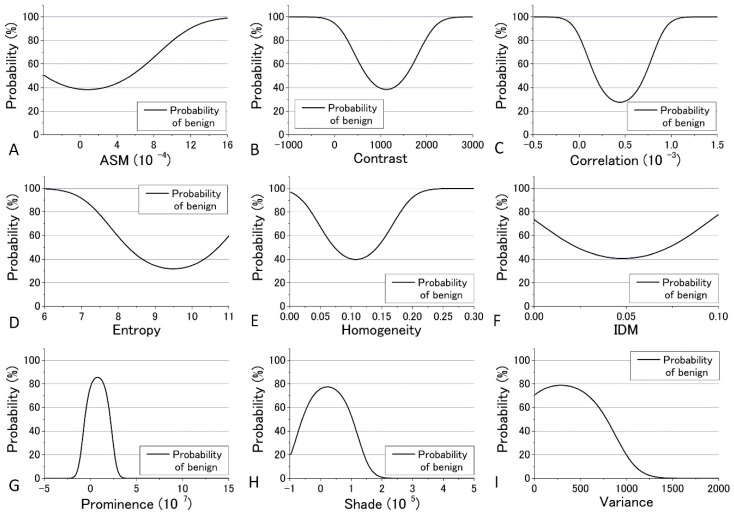
Probability of benign plotted against the parameter values for the distance *d* = 10 at 488 nm excitation.

In the previous analysis, DIF was calculated using the average of the parameters obtained from the four images which contain 48 raw images. The calculated values of DIF for the nine GLCM parameters are shown in Table S6. To further utilize the parameters obtained by the GLCM analysis, we assessed them by taking the dispersion of the distribution of the parameters into account. The mean and distribution (=dispersion) were fitted with a Gaussian distribution as shown in Figure 9 and Figure 10 for all nine parameters. These figures show that the values of the parameters are widely distributed and depict that the degree of overlap between the nevus and MM cells are different among the parameters. Using the distribution function, the probability of benign (defined by B = Benign/(Benign + Malignant)) was then calculated and plotted against the parameter values for the distance *d* = 10. The results are shown in Figure 12 and Figure 13.

We tried to evaluate the appropriateness of assigning either nevus or MM to cells using another simple discrimination level for each. For this, we adopted a “clearness discrimination parameter” (DISC value), that is defined by the following equation for each parameter using the probability of benign (B) shown in Figure 12 and Figure 13:

DISC = {(parameter at 65% of B) − (parameter at 35% of B)}/{(parameter at 65% of B)/2 + (parameter at 35% of B)/2}
(13)

Here, “35% of B” means the value of the corresponding parameter at 35% of its maximum intensity. Usually, 90% and 10% are used for the level of discrimination or for steepness evaluation for the distribution curve; for some of the parameters, however, the B value did not reach 10% for any parameter in the present analysis. The smaller the DISC value, the more accurate the discrimination. The calculated results are shown in Table 1 and Table 2 in ascending order of DISC value at 405 nm and 488 nm excitation, respectively. In 405 nm excitation, the DISCs for nevus and MM, Entropy, Homogeneity, and Variance, were conspicuously small, which was expected to be effective for that discrimination (Table 1). Additionally, in the GLCM calculation result at 488 nm excitation, the DISCs for nevus and MM, Entropy, Prominence, and Correlation, were the three smallest (Table 2). Thus, there were differences in effective parameters depending on excitation wavelength. This can be well explained in terms of the sensitivity of the signal intensity to the components in the cells, namely melanin and porphyrin, due to the differences in the absorption cross section between them.

The ROC curve is drawn by plotting the true positive ratio (sensitivity) against the false positive ratio as the threshold parameter goes through the measuring range. We plotted the ROC curves based on Figure 9 and Figure 10, and the curves took on distinct shapes depending on the SDs and the difference of the means of the two cell types. (1) The difference of mean values of both cell types were near the SDs, as seen in the case of ASM, Contrast, Entropy, Homogeneity, IDM and Variance at 405 nm excitation and Entropy at 488 nm excitation. In this case, the ROC curves rose quite precipitously, gradually decreased their slopes and got almost horizontal at the end, however, always kept convex upward. (2) SDs of the parameters for the nevus cells were large enough that the distribution for them spread to cover the main part of the distribution for MM cells, as seen in the case of Correlation, Prominence and Shade at 405 nm excitation and Correlation at 488 nm excitation. In this case, the ROC curve rose precipitously, however, after a short time, decreased its slopes to nearly horizontal, and again got steep at the end. This is because at first, only nevus cells were considered as positive and MM cells were not included within threshold, and hence the false positive was kept very low. As the threshold parameter passed through the main part of MM cells, the cumulating values were mainly due to MM cells, resulting in the horizontal shape. After the threshold-parameter passed the main part of MM cells, nevus cells mainly count for the positives. (3) SDs of the parameters for the MM cells were large enough that the distribution for them spread to cover the main part of the distribution for nevus cells, as seen in the case of Prominence, Shade and Variance at 488 nm excitation. In this case, the ROC curves first went horizontally, however, after a short time, rose up very steeply and got to nearly horizontal at the end. The reason for this behavior is reverse to (2).

**Figure 14:**
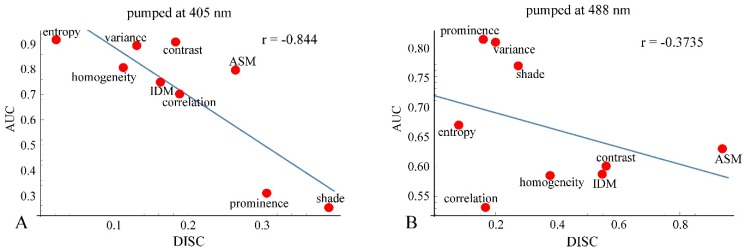
Correlation diagrams between AUC and DISC at 405 nm excitation (**A**) and at 488 nm excitation (**B**).

As mentioned previously, analyses of ROC and DIF agreed quite well at both 405 nm and 488 nm. However, there were some differences between the analyses results of ROC or DIF and DISC, especially at 488 nm excitation. The correlation diagrams between AUC and DISC at 405 nm and at 488 nm excitation are shown in Figure 14. The correlation coefficient at 405 nm excitation was −0.844, indicating a strong correlation, and at 488 nm excitation, it was −0.3735, showing a weak correlation.

Analyses based on DIF, ROC, and DISC for evaluating the diagnostic ability of GLCM-parameters showed little difference between 405 nm and 488 nm excitation. The difference between the calculation results for experiments performed at 405 nm and 488 nm excitation can be explained in more detail as follows.

The molar extinction coefficients of melanin at 405 nm and 488 nm in the literature are approximately 2500 and 1500/mol/cm, respectively [41]. In contrast, the molar extinction coefficient of hemoglobin at 405 nm and 488 nm is about 275,000 and 16,000/mol/cm, respectively. At both wavelengths, the molar extinction coefficient of hemoglobin is high, but the absolute values are quite different. The extinction coefficient of hemoglobin at 488 nm is 10 times larger than that of melanin, while the coefficient of hemoglobin at 405 nm is 100 times larger than that of melanin. The amount of melanin contained in the cell slice is 10 times greater than that of hemoglobin. Thus, it might be possible to assess the state or degree of transformation of the skin tissue by monitoring with GLCM analysis of 488 nm pump images. It may be possible that at 488 nm excitation, Prominence and Entropy detected the changes of melanin distribution induced by the transformation of cells.

At 405 nm excitation, Homogeneity and Entropy might detect the changes in hemoglobin distribution induced by cellular transformation.

We defined DISC using 65% and 35% DIF values instead of 10% and 90% values, which are commonly used. This might diminish the performance of DISC analysis and might provide some discordance with ROC analysis (Figure 14A,B). We devised the DISC analysis as a simple and versatile method to evaluate the diagnostic abilities of the GLCM parameters. Applying this to a wider range of data will hopefully improve the performance of DISC analysis.

In view of the purpose of this study, it can be concluded that melanin observation by 488 nm excitation is more suitable for the determination of cancerous tumors, so the conclusion is that Entropy and Prominence at 488 nm excitation are suitable for benign-malignancy determination. By utilizing 405 nm excitation, we may be able to study the effect of cancerous tumors on the hemoglobin-containing tissues, such as muscle attached to the sample slices. In this case, Homogeneity and Entropy can be used for benign-malignancy determination. This may correspond to the morphological change in hemoglobin distribution induced by cellular tumorigenesis. This means that the spatial distribution of cancerous tumors can be investigated through hemoglobin.

## 5. Conclusions

Label-free confocal photothermal (CPT) microscopy combined with a texture analysis method was utilized for the first time to investigate benign-malignancy determination in mouse skin cells. A Grey Level Cooccurrence Matrix (GLCM) method for texture analysis was applied to the CPT images of malignant melanoma (MM) to study the differences in intracellular super-resolved structural properties between MMs and nevus. We first applied DIF analysis and obtained the results that Entropy, Contrast, and Variance were most suited for the discrimination between MM and nevus when 405 nm excitation was used, and Prominence, Variance, and Shade were most suited at 488 nm excitation. Analysis based on ROC gave the same results. DISC, which we introduced as a new discrimination parameter, suggested Entropy, Homogeneity, and Variance were best at 405 nm excitation, and Entropy, Prominence, and Correlation were best at 488 nm excitation. Observation at 405 nm excitation might detect the morphological change in hemoglobin distribution induced by cellular transformation, whereas observation at 488 nm excitation caught the change of melanin distribution. The differences in effective parameters due to differences in excitation wavelength can be well explained in terms of the sensitivity of the signal intensity to the components in the cells, namely melanoma and porphyrin (hemoglobin), due to the difference in the absorption cross section between them.

## Figures and Tables

**Table 1:** The clearness discrimination parameter (DISC) value for the nine GLCM parameters (405 nm excitation).

Parameter	65%	35%	DISC
Entropy	8.34	8.52	0.021
Homogeneity	0.121	0.108	0.112
Variance	913	1040	0.13
IDM	0.0598	0.0508	0.162
Contrast	142	1700	0.182
Correlation	3.04	2.52	0.188
ASM	3.98	3.06	0.264
Prominence	5.25 × 10^8^	3.86 × 10^8^	0.306
Shade	256,900	173,000	0.39

**Table 2:** DISC value for the nine GLCM parameters (488 nm excitation).

Parameter	40%	60%	DISC
Entropy	8.64	7.97	0.080
Prominence	2.34 × 10^7^	1.99 × 10^7^	0.159
Correlation	6.47	7.65	0.166
Variance	856	700	0.200
Shade	117,000	888	0.274
Homogeneity	0.107	0.157	0.378
IDM	0.0480	0.0842	0.547
Contrast	968	544	0.560
ASM	2.60 × 10^−4^	7.20 × 10^−4^	0.939

## Supplementary Materials

The following are available online at http://www.mdpi.com/2306-5354/5/3/67/s1, Table S1: Results of calculated GLCM diagnosis method (405 nm excitation), Table S2: Results of calculated GLCM diagnosis method (488 nm excitation), Table S3: Order of the DIF values among the nine parameters (405 nm excitation), Table S4: Order of the DIF values among the nine parameters (488 nm excitation), Table S5: Calculated values of DIF for the nine GLCM parameters (average of 48 data from 48 images), Table S6: Calculated values of DIF for the nine GLCM parameters (average of data from 48 images).
