# Supplementary material for: Label-Free Imaging of Melanoma with Confocal Photothermal Microscopy: Differentiation between Malignant and Benign Tissue
Source: Bioengineering (Basel). 2018 Aug 15;5(3):67. doi: 10.3390/bioengineering5030067 (PMC6163989; doi:10.3390/bioengineering5030067)
Supplement: Supplementary file 1 [file bioengineering-05-00067-s001.pdf]

Table 1. Results of calculated GLCM diagnosis method (405 nm excitation).

Parameter	SN	SP	AC	LR+	LR−	PPV	NPV
ASM	0.8017	0.7630	0.7823	3.382	0.2600	0.7717	0.7938
IDM	0.6972	0.8224	0.7599	3.927	0.3681	0.7970	0.7310
Contrast	0.8350	0.8100	0.8225	4.394	0.2038	0.8146	Accuracy

Table 2. Results of calculated GLCM diagnosis method (488 nm excitation).

Parameter	SN	SP	AC	LR+	LR−	PPV	NPV
ASM	0.722	0.461	0.592	1.34	0.603	0.573	0.624
Contrast	0.826	0.370	0.598	1.31	0.471	0.567	0.680
Correlation	--	--	--	--	--	--	--
Entropy	0.706	0.547	0.627	1.56	0.537	0.609	0.651
Homogeneity	0.813	0.356	0.585	1.26	0.526	0.558	0.656
IDM	0.811	0.350	0.580	1.25	0.540	0.555	0.649
Prominence	0.683	0.954	0.819	14.8	0.332	0.937	0.751
Shade	0.592	0.908	0.750	6.42	0.450	0.865	0.690
Variance	0.647	0.858	0.753	4.57	0.411	0.820	0.709

Table 3. Order of the DIF values among the nine parameters (405 nm excitation).

d	ASM	Contrast	Correlation	Entropy	Homogeneity	IDM	Prominence	Shade	Variance
1	4	8	3	2	6	7	9	5	1
2	5	4	3	2	6	7	9	8	1
3	3	6	4	2	5	7	9	8	1
4	3	4	5	2	6	7	9	8	1
5	3	4	7	2	5	6	8	9	1
6	4	3	7	2	5	6	8	9	1
7	4	3	7	2	5	6	8	9	1
8	6	3	7	1	5	4	8	9	2
9	6	3	7	1	5	4	8	9	2
10	4	2	7	1	5	6	8	9	3

Table 4. Order of the DIF values among the nine parameters (488 nm excitation).

d	ASM	Contrast	Correlation	Entropy	Homogeneity	IDM	Prominence	Shade	Variance
1	7	4	5	6	8	9	1	3	2
2	7	5	4	6	8	9	1	3	2
3	6	7	5	4	8	9	1	3	2
4	6	9	4	5	8	7	1	3	2
5	5	7	6	4	9	8	1	3	2
6	5	9	6	4	8	7	1	3	2
7	5	9	8	4	7	6	1	3	2
8	5	7	6	4	8	9	1	3	2
9	5	8	9	4	7	6	1	3	2
10	5	6	9	4	7	8	1	3	2

Table 5. Calculated values of DIF for the nine GLCM parameters (average of 48 data from 48 images).

d	ASM	Contrast	Correlation	Entropy	Homogeneity	IDM	Prominence	Shade	Variance
1	1.274	0.589	1.423	1.504	0.623	0.602	0.2413	0.6622	1.832
2	1.525	1.581	1.659	1.799	1.408	1.369	0.0372	0.4588	1.860
3	1.376	1.059	1.256	1.641	1.065	1.000	0.0783	0.5325	1.844
4	1.419	1.225	1.205	1.685	1.169	1.066	0.0692	0.3591	1.835
5	1.452	1.365	1.195	1.708	1.291	1.206	0.1987	0.1744	1.827
6	1.465	1.486	1.212	1.734	1.332	1.240	0.2988	0.0120	1.814
7	1.498	1.604	1.256	1.797	1.430	1.329	0.3670	0.1179	1.802
8	1.524	1.710	1.308	1.820	1.658	1.665	0.4063	0.2081	1.802
9	1.543	1.792	1.347	1.854	1.750	1.789	0.4205	0.2625	1.816
10	1.555	1.847	1.350	1.879	1.512	1.386	0.4054	0.2837	1.836

Table S6. Calculated values of DIF for the nine GLCM parameters (average of data from 48 images).

d	ASM	Contrast	Correlation	Entropy	Homogeneity	IDM	Prominence	Shade	Variance
1	0.334	0.603	0.415	0.372	0.175	0.127	1.374	1.052	1.268
2	0.365	0.474	0.507	0.429	0.049	0.005	1.412	1.063	1.268
3	0.376	0.367	0.428	0.473	0.042	0.015	1.451	1.074	1.269
4	0.407	0.074	0.614	0.528	0.112	0.123	1.452	1.08	1.27
5	0.421	0.11	0.291	0.549	0.082	0.094	1.477	1.084	1.271
6	0.449	0.061	0.336	0.582	0.217	0.228	1.465	1.088	1.272
7	0.456	0.206	0.279	0.606	0.282	0.285	1.453	1.094	1.273
8	0.462	0.327	0.399	0.61	0.26	0.248	1.459	1.102	1.273
9	0.472	0.248	0.084	0.627	0.317	0.325	1.479	1.108	1.273
10	0.473	0.367	0.137	0.628	0.315	0.312	1.472	1.121	1.273
